# Selected HLA-B allotypes are resistant to inhibition or deficiency of the transporter associated with antigen processing (TAP)

**DOI:** 10.1371/journal.ppat.1007171

**Published:** 2018-07-11

**Authors:** Jie Geng, Anita J. Zaitouna, Malini Raghavan

**Affiliations:** Department of Microbiology and Immunology, Michigan Medicine, University of Michigan, Ann Arbor, Michigan, United States of America; University of California, UNITED STATES

## Abstract

Major histocompatibility complex class I (MHC-I) molecules present antigenic peptides to CD8^+^ T cells, and are also important for natural killer (NK) cell immune surveillance against infections and cancers. MHC-I molecules are assembled via a complex assembly pathway in the endoplasmic reticulum (ER) of cells. Peptides present in the cytosol of cells are transported into the ER via the transporter associated with antigen processing (TAP). In the ER, peptides are assembled with MHC-I molecules via the peptide-loading complex (PLC). Components of the MHC-I assembly pathway are frequently targeted by viruses, in order to evade host immunity. Many viruses encode inhibitors of TAP, which is thought to be a central source of peptides for the assembly of MHC-I molecules. However, human MHC-I (HLA-I) genes are highly polymorphic, and it is conceivable that several variants can acquire peptides via TAP-independent pathways, thereby conferring resistance to pathogen-derived inhibitors of TAP. To broadly assess TAP-independent expression within the HLA-B locus, expression levels of 27 frequent HLA-B alleles were tested in cells with deficiencies in TAP. Approximately 15% of tested HLA-B allotypes are expressed at relatively high levels on the surface of TAP1 or TAP2-deficient cells and occur in partially peptide-receptive forms and Endoglycosidase H sensitive forms on the cell surface. Synergy between high peptide loading efficiency, broad specificity for peptides prevalent within unconventional sources and high intrinsic stability of the empty form allows for deviations from the conventional HLA-I assembly pathway for some HLA-B*35, HLA-B*57 and HLA-B*15 alleles. Allotypes that display higher expression in TAP-deficient cells are more resistant to viral TAP inhibitor-induced HLA-I down-modulation, and HLA-I down-modulation-induced NK cell activation. Conversely, the same allotypes are expected to mediate stronger CD8^+^ T cell responses under TAP-inhibited conditions. Thus, the degree of resistance to TAP inhibition functionally separates specific HLA-B allotypes.

## Author summary

Human leukocyte antigen (HLA) class I molecules present pathogen-derived components (peptides) to cytotoxic T cells, thereby inducing the T cells to kill virus-infected cells. A complex cellular pathway involving the transporter associated with antigen processing (TAP) is typically required for the loading of peptides onto HLA class I molecules, and for effective anti-viral immunity mediated by cytotoxic T cells. Many viruses encode inhibitors of TAP as a means to evade anti-viral immunity by cytotoxic T cells. In humans, there are three sets of genes encoding HLA class I molecules, which are the *HLA-A*, *HLA-B and HLA-C* genes. These genes are highly variable, with thousands of allelic variants in human populations. Most individuals typically express two variants of each gene, one inherited from each parent. We demonstrate that about 15% of tested HLA-B allotypes have higher resistance to viral inhibitors of TAP or deficiency of TAP, compared to other HLA-B variants. HLA-B allotypes that are more resistant to TAP inhibition are expected to induce stronger CD8^+^ T cell responses against pathogens that inhibit TAP. Thus, unconventional TAP-independent assembly pathways are broadly prevalent among HLA-B variants. Such pathways provide mechanisms to effectively combat viruses that evade the conventional TAP-dependent HLA-B assembly pathway.

## Introduction

MHC-I molecules play a pivotal role in immune surveillance of intracellular pathogens by presenting antigenic peptides to cytotoxic T cells (CTL). They also function to regulate natural killer (NK) cell activity by engaging NK cell receptors including KIR3DL1 [1], KIR2DL1/2/3 [2], CD94-NKG2A [3] and KIR3DS1 [4, 5]. MHC-I molecules have strong influences on disease progression in a number of infectious diseases and cancers [6, 7]. In many cases, the peptide-binding characteristics of individual MHC-I proteins are the major factor that determines immune control of diseases, but other characteristics of the MHC-I molecules, such as those relating to variations in the assembly and stability of individual MHC-I molecules, may also have an influence on disease outcomes.

Intracellular proteins are generally degraded into peptide fragments by the ubiquitin-proteasome system [8]. Peptides that bind MHC-I molecules are typically translocated into the ER lumen by the transporter associated with antigen processing (TAP) and then loaded onto MHC-I molecules with the help of other components of the peptide-loading complex (PLC), including tapasin, calreticulin and ERp57 [9]. Empty forms of MHC-I molecules are less thermostable than peptide-filled versions of MHC-I molecules [10–12]. ER quality control, including interactions with the PLC and calreticulin-mediated retrieval [13], contributes to the intracellular retention of empty forms of MHC-I molecules. Additionally, tapasin and the tapasin-related protein (TAPBPR) edit and proofread the MHC-I peptide repertoire by replacing suboptimal low affinity peptides with optimal high affinity peptides [14–20] that can mediate more durable CD8^+^ T cell responses. In general, an intact PLC is essential for efficient peptide assembly with MHC-I molecules and successful ER quality control. However, individual MHC-I allotypes are known to have different requirements for each component of the PLC. For example, high cell surface expression of some human MHC-I (HLA-I) allotypes is observed in tapasin-deficient cells, whereas other allotypes are poorly expressed [16, 21, 22]. There are known differences in steady state binding of HLA-I molecules to TAP [23]. There are also known allomorph-specific differences in proteasome-dependence [24].

TAP is thought to be the major cellular source of peptide for assembly of most MHC-I molecules. In TAP-deficient cells, MHC-I cell surface expression is generally severely compromised [25–27]. Many viruses down-regulate or inhibit TAP to evade CTL responses [28, 29]. In previous *in vitro* studies, we found that HLA-B allotypes display a hierarchy of refolding efficiencies and thermostabilities of heavy chains with β2-microglobulin (β2m) in the absence of peptide [12, 22], suggesting distinct intrinsic stabilities of empty forms of HLA-B. Molecular dynamics stimulations have also indicated that empty forms of some HLA-B molecules are more disordered than others [30–32]. Hein et. al. have shown that increasing intrinsic stability of H2-K^b^-β2m complex by connecting the α1 and α2 helices with a disulfide bond close to the F-pocket, allowed suboptimally loaded forms of H2-K^b^ to bypass all cellular quality control steps in TAP-deficient cells [33]. Thus, in the trafficking process, the stability of empty heavy chain-β2m complexes is a key factor that determines the fate of MHC-I molecules in TAP-deficient cells. These findings raised the question of whether empty and suboptimally loaded forms of the more thermostable HLA-B allotypes can bypass ER quality control, traffic to the cell surface and maintain an increased steady-state presence there. Additionally, there can be influences of MHC-I peptide-binding specificities upon HLA-I cell surface expression levels under different conditions. It is known that peptides containing proline at the P2 or P3 position are poorly transported by TAP [34, 35], making it possible that MHC-I allotypes with these binding preferences (for example, HLA-B allotypes of the B7 supertype [36]) are more reliant on additional/alternate sources of peptide, and will have reduced sensitivity to TAP inhibition. Based on these observations, we hypothesized therefore that cell surface expression of MHC-I molecules would be differently dependent on TAP (the major source of MHC-I peptides), based on the intrinsic stabilities of their empty forms and peptide-binding specificity differences. As described below, our studies revealed differential expression levels of HLA-B allotypes on the surface of TAP-deficient and TAP-inhibited cells. Intrinsic stability of the empty form as well as peptide-binding preferences determine cell surface expression levels under TAP-deficiency conditions. Furthermore, we showed that cells expressing HLA-B molecules with Bw4 epitopes that are resistant to inhibition of TAP are more resistant to the activation of KIR3DL1^+^ NK cells under TAP-inhibited conditions. Together, our findings indicate that HLA-I molecules have evolved to assemble via distinct pathways, which are allotype dependent, as a way to counter pathogen evasion strategies that target the conventional assembly pathway.

## Results

### Variable HLA-B cell surface expression in TAP-deficient cells

**Fig 1:**
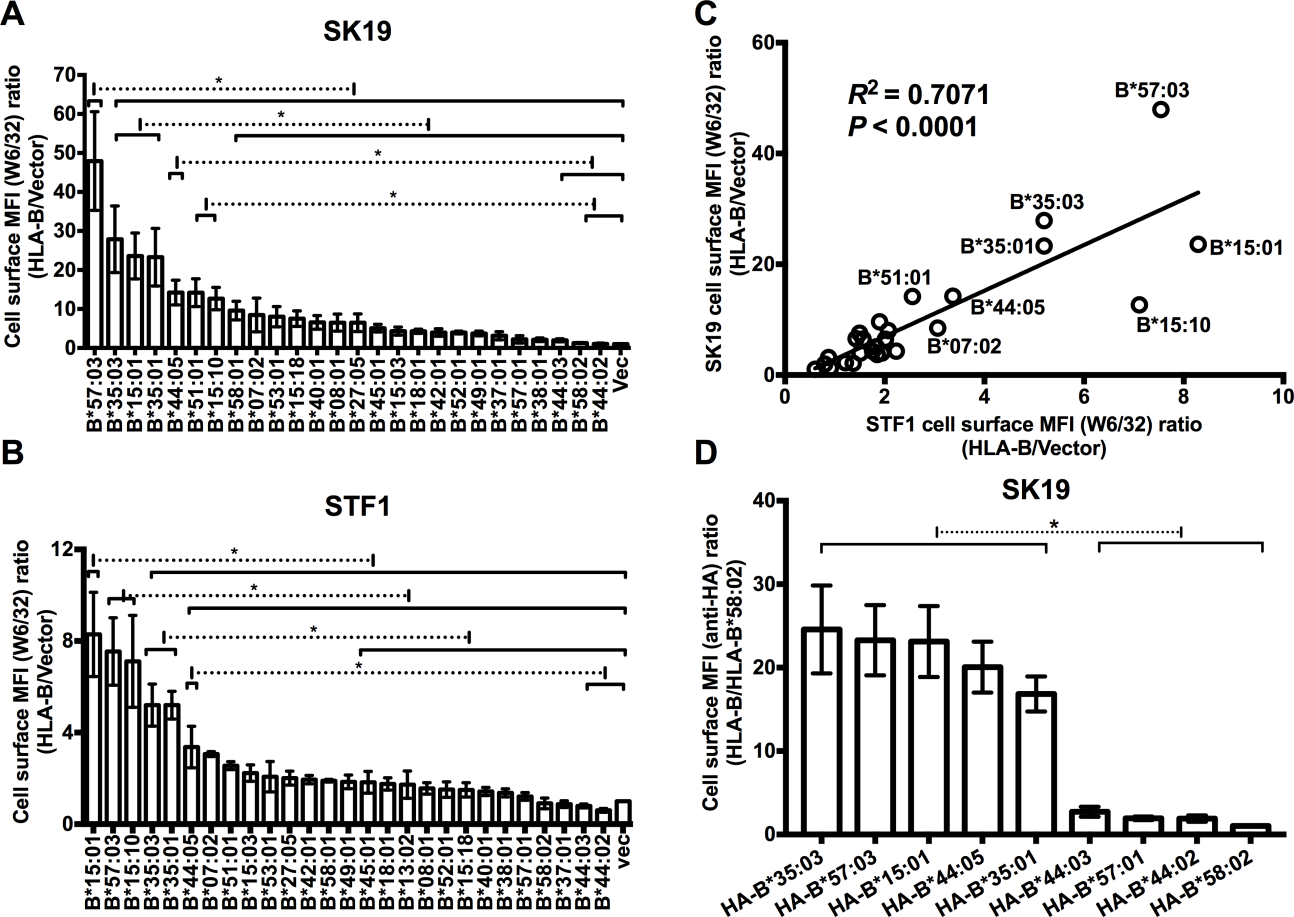
Allotype-dependent variations in cell surface HLA-B expression in TAP-deficient cells. (A and B) Cell surface HLA-B levels in SK19 (TAP1-deficient) or STF1 (TAP2-deficient) cells infected with retroviral constructs encoding indicated HLA-B were expressed as mean fluorescence intensity (MFI) ratios relative to those obtained for infections with an empty retroviral vector lacking HLA-B (vec). Data are derived from 4–13 (A) or 2–9 (B) flow cytometric measurements with the W6/32 antibody following 2–5 (A) or 1–4 (B) separate retroviral infections. (C) The MFI ratios for HLA-B allotypes from SK19 cells (Panel A) correlate with those from STF1 cells (Panel B). (D) SK19 cells were also infected with retroviruses encoding HA-tagged versions of HLA-B (HA-HLA-B). Cell surface expression levels of HLA-B molecules were tested by flow cytometry after staining with anti-HA. Data are derived from 3 measurements following one infection. Significant differences are indicated (with an asterisk) on the graph (*P*<0.05). Statistical significance is based on an ordinary one-way ANOVA analysis with Fisher’s LSD test.

In TAP-deficient cells, where the majority of peptides are prevented from entering ER, most HLA-I molecules are empty or suboptimally loaded and HLA-I cell surface expression is generally significantly reduced [25–27]. We expected that when peptide supply is highly deficient in the ER, allotypes with higher intrinsic stabilities of their empty forms might have a better chance to bypass the quality control system as empty molecules or after being loaded with suboptimal peptides to become expressed on the cell surface. To examine whether HLA-B allotypes differ in their abilities to become expressed on the surface of TAP-deficient cells, several HLA-B allotypes that occur at the highest frequencies in United States populations were expressed in the TAP1-deficient human melanoma cell line SK-mel-19 (SK19) [37] or in a TAP2-deficient human fibroblast cell line STF1 [38] using the previously described retroviral infection method [22, 39]. Cell surface expression of HLA-B allotypes was analyzed by flow cytometry after staining with W6/32, which recognizes different HLA-I allotypes with similar affinities. HLA-B allotypes showed large variations in cell surface expression in SK19 cells and STF1 cells (Fig 1A and 1B). Cell surface expression of HLA-B*57:03, B*35:03, B*15:01, B*35:01, and B*15:10 was over 10-fold higher than the cell surface expression of the endogenous HLA-I of SK19 cells and over 5-fold higher than the cell surface expression of the endogenous HLA-I of STF1 cells (Fig 1A and 1B). Cell surface expression of B*44:03, B*58:02 and B*44:02 was very low or undetectable in STF1 cells, and less than two-fold above endogenous HLA-I cell surface expression in SK19 cells (Fig 1A and 1B). Other HLA-B allotypes showed intermediate phenotypes (Fig 1A and 1B).

In general, there was poor correlation between exogenous HLA-I cell surface expression assessed by flow cytometry (Fig 1A and 1B) and total cellular expression assessed by immunoblotting analyses for HLA-I heavy chains (S1A and S1B Fig). For SK19 cells or STF1 cells with HLA-B that were detectable at low or high levels on the cell surface, overexpression of exogenous HLA-B molecules did not induce any consistent unfolded protein response (UPR) compared with vector-infected cells, as assessed by immunoblots for BiP (S2 Fig), induction of which is an UPR indicator [40]. There was a strong correlation between HLA-B cell surface expression levels in STF1 cells and those in SK19 cells (Fig 1C), suggesting that the HLA-B cell surface expression differences were not cell dependent, but rather were TAP-deficiency dependent. Supporting the latter possibility, we have previously shown small differences in the cell surface expression of the HLA-B allotypes in TAP-expressing cells such as a CD4^+^ T cell line, CEM [22]. To verify that the measured W6/32 signals in SK91 and STF1 cells reflect the intended HLA-B signals rather than any other possible signals, HA-tagged versions of selected HLA-B that were detectable at high or low levels in SK19 and STF1 cells were constructed and expressed in SK19 cells by retroviral infection. An antibody against the HA epitope tag was used to test cell surface or total HA-tagged HLA-B (HA-HLA-B) expression. The HA-HLA-B versions maintained the same expression phenotypes as their untagged counterparts (Fig 1D, S1C Fig).

### Differential effects of TAP1 restoration and blockade on HLA-B cell surface expression

**Fig 2:**
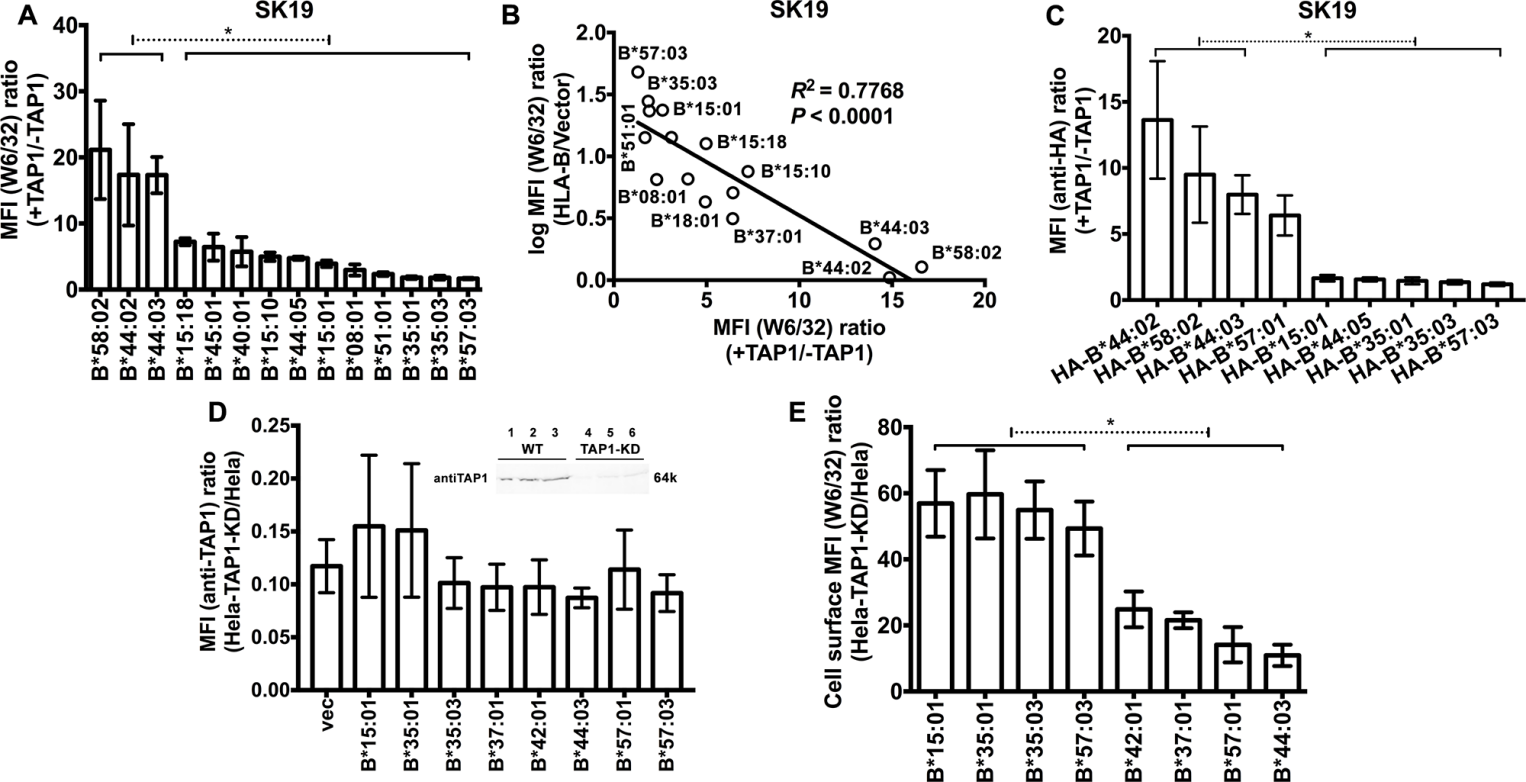
TAP1-dependencies of HLA-B expression. (A) Cells from one infection from Fig 1A were subsequently infected with a TAP1-encoding retrovirus. The MFI ratios in the presence and absence of TAP1 (+TAP1/-TAP1) were calculated for each HLA-B–expressing cell line (n = 4 analyses from one infection). (B) Correlation between surface HLA-B expression in SK19 cells (calculated from Fig 1A) and their +TAP1/-TAP1 MFI ratios (calculated from Fig 2A). (C) Cells from the infections shown in Fig 1D were subsequently infected with a TAP1-encoding retrovirus. The MFI ratios in the presence and absence of TAP1 (+TAP1/-TAP1) were calculated for each HA-HLA-B–expressing cell line (n = 4 analyses from one infection). (D) Parental and TAP1-knockdown Hela cells (Hela-TAP1-KD) were assessed by immunoblotting with the anti-TAP1 antibody 148.3 (inset panel; 5, 10 or 20 μg of cell lysate was loaded in each lane) and infected with retroviruses encoding HLA-B allotypes or a control retrovirus (vector). TAP1 expression levels were measured by flow cytometry after intracellular staining with 148.3 antibody. (E) HLA-B expression levels at the surface of Hela or Hela-TAP1-KD cells were measured after W6/32 staining. The MFI ratios (Hela-TAP1-KD/Hela) were calculated for each HLA-B–expressing cell line (n = 6–7 measurements from three separate infections of Hela or Hela-TAP1-KD cells with retroviruses encoding indicated HLA-B). Significant differences are indicated (with an asterisk) on the graph (*P*<0.05). Statistical significance is based on an ordinary one-way ANOVA analysis with Fisher’s LSD test.

To confirm varying TAP-dependencies of HLA-B cell surface expression, we examined TAP1-mediated cell surface induction of HLA-B molecules following further infection of selected SK19-HLA-B cell lines with a TAP1-encoding retrovirus (S3A Fig). There was an inverse correlation between the extent of TAP1-mediated induction (+TAP1/-TAP1) and cell surface expression under TAP1-deficiency conditions (Fig 2A and 2B). TAP1 expression was also reconstituted in SK19 cells expressing the HA-HLA-B (S3B Fig). There was again an inverse correlation between the extent of TAP1-mediated induction (+TAP1/-TAP1) and cell surface expression under TAP1-deficiency conditions (Fig 2C). To validate the TAP-dependency results, TAP1 was knocked-down in a TAP-sufficient easily–transfectable cell line, Hela. TAP1-knock down (KD) or parent Hela cells were infected with retroviruses encoding selected HLA-B allotypes that were detectable at high, low or intermediate levels in TAP1 and TAP2-deficient cells (as shown in Fig 1A and 1B). The allotypes expressed at high levels in SK19 and STF1 cells were down-modulated to a lesser extent by TAP1 knockdown compared to the HLA-B allotypes expressed at low levels in SK19 and STF1 cells (Fig 2D and 2E), consistent with the conclusion from TAP induction experiments. Thus, HLA-B allotypes have differential resistance to inhibition of TAP (RIT) phenotypes.

### Mechanisms determining TAP-independent expression

Higher intrinsic stability of the empty form, measured for many tapasin-independent allotypes [12, 22], would also favor a higher efficiency of peptide loading and thus cell surface expression under TAP-deficiency conditions. Zernich et. al. [41] attributed the advantage of B*44:05 cell surface expression under conditions of limiting peptide supply to the high peptide loading efficiency of nascent B*44:05, which also causes its tapasin independency [16, 22, 41]. The structural similarities between the F-pockets of B*44:05 and B*57:03 (the presence of Y116) might confer efficient peptide loading to both allotypes, while residue 116 is a D in B*44:02 and S in B*57:01. Differences in peptide loading efficiencies between B*57:03 and B*57:01 could explain the differences in tapasin- and TAP-dependencies of these two closely-related allotypes, which differ only in the F-pocket regions, at positions 114 and 116.

**Fig 3:**
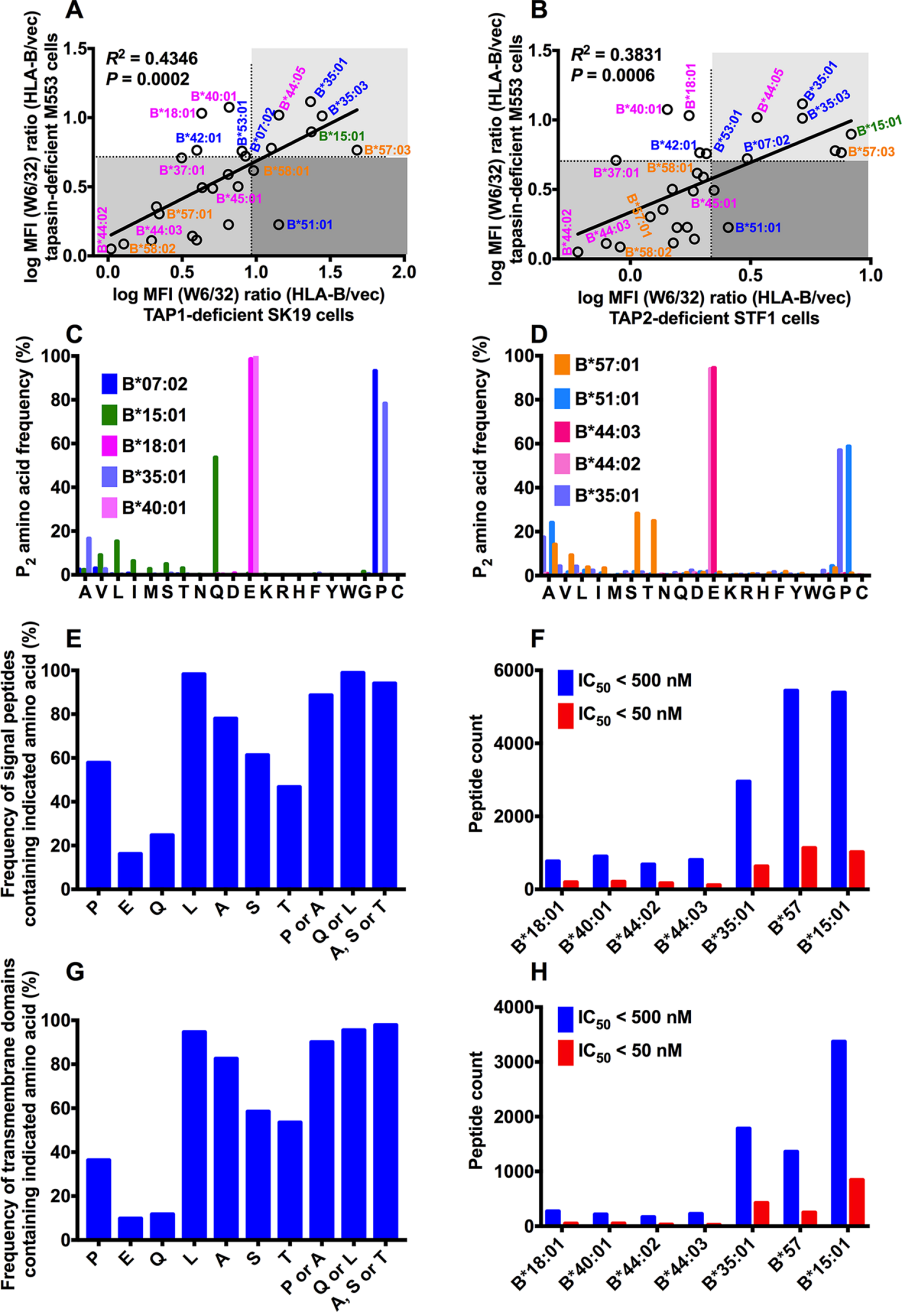
Mechanisms determining TAP-independent HLA-B surface expression. The TAP1 dependencies (A) and TAP2 dependencies (B) of HLA-B allotypes are partly correlated with their tapasin dependencies. The MFI ratios of HLA-B allotypes (derived from Fig 1A or 1B) were used as indexes of TAP1 and TAP2 dependencies and MFI ratios of HLA-B allotypes (derived from Fig 1A of Ref. 22) were used as index of tapasin dependencies. HLA-B supertypes are color-coded, B7, blue; B44, pink; B62, green; B58, orange. (C and D) The anchor residue preferences at P_2_ based on peptide sequences mined from two recent mass spectrometric studies (Ref. 42 and 43 respectively). In A-D, alleles belonging to the same supertype are color-coded. (E) Frequencies of indicated amino acids at the N-termini (excluding the last 6 residues at the C-terminus which cannot be P_2_ for any epitope) of known human signal peptide sequences obtained from www.signalpeptide.de. (F) Signal peptide sequences were used to predict potential 9-mer epitopes for the indicated HLA-B, using NetMHC 4.0. For each allele, the number of peptides with predicted IC_50_ values < 500 nM and 50 nM are shown. (G and H) Similar to E and F, but using human transmembrane domain sequences obtained from ftp://ftp.ncbi.nih.gov/repository/TMbase/, to estimate the frequencies of occurrence of indicated amino acids, excluding the last 6 residues at the C-terminus (G) and the predicted number of epitopes for each indicated HLA-B (H).

While there is a partial positive correlation between TAP-dependence and tapasin-dependence of HLA-B cell surface expression (Fig 3A and 3B), some allotypes are clear outliers. Individual HLA-B allotypes have different dependencies on TAP and tapasin. Some highly tapasin-independent allotypes such as B*18:01 and B*40:01, both members of the B44 supertype (pink, favoring peptides containing glutamic acid at position 2 (P_2_)), are more TAP dependent. Some highly tapasin-dependent allotypes such as B*51:01, a member of the B7 supertype (blue, similar to B*35:01 and B*35:03, favoring peptides containing proline at P_2_), are less TAP dependent (Fig 3A and 3B). These findings indicate that, the underlying mechanisms of TAP-independence and tapasin-independence are not fully overlapping.

Recent mass spectrometric studies have identified large numbers of HLA-I peptidomes for different allotypes. Comparisons of the anchor residue preferences based on peptide sequences mined from two recent datasets [42, 43] revealed that RIT allotypes generally have higher P_2_ diversity than several other non-RIT HLA-B (Fig 3C and 3D), which would also favor selection of TAP-independent peptide from unconventional sources. It is noteworthy that there is a strict conservation of P_2_ among members of the B44 supertype (including B*44:02, B*44:03, B*18:01 and B*40:01 (pink; Fig 3C and 3D)) compared to members of the B7 supertype (including B*35:01, B*51:01 and B*07:02 (blue; Fig 3C and 3D)). Glutamic acid is stringently conserved as a P_2_ anchor among these members of the B44 supertype, whereas proline, alanine, and other residues occurring at lower frequencies, are found as P_2_ anchors among members of the B7 supertype (based on data from Ref. 42 (Fig 3C) and 43 (Fig 3D)). B*15:01, another allotype with high RIT, also displays high sequence diversity at the peptide P_2_ position (53% Q, 15% L, 9% V, 6%I, 5% S, 12% other) (based on data from Ref. 42; Fig 3C). Although a large peptidome dataset is not available for HLA-B*57:03, recent B*57:01 peptidome data indicate high diversity at the peptide P_2_ position (based on data from Ref. 43; Fig 3D). Structural similarities between the B pockets of B*57:01 and B*57:03 (the P_2_ binding pocket) predict a high P_2_ diversity for peptides that bind B*57:03, similar to B*57:01.

Based on prior studies [44–47], signal peptides and hydrophobic peptides are expected to be a TAP-independent source of MHC-I peptides. We first examined the prevalence of anchor residues for TAP-dependent and RIT allotypes within human signal sequence datasets. Within known human signal peptide sequences (www.signalpeptide.de), N-terminal prolines and alanines (excluding the last 6 residues at the C-terminus, which cannot be a P_2_ residue for any HLA-I epitope), preferred anchor residues for the B7 supertype, are significantly more prevalent than N-terminal glutamic acid, the preferred anchor residue for the B44 supertype (Fig 3E). The low prevalence of glutamic acid within signal sequences could explain why the TAP-dependence phenotypes of B*18:01 does not mirror its high tapasin-independence and stability [12, 22]. Conversely, the higher prevalence of proline/alanine within signal sequences could explain why the TAP-dependence phenotype of B*51:01 is less stringent than predicted by its strong tapasin-dependence and lower stability [12, 22]. Preferred P_2_ residues for other RIT allotypes, such as B*57:03 (A/S/T) and B*15:01 (Q/L), are also highly represented within the N-termini of signal peptide sequences (Fig 3E). Further, using the NetMHC algorithm [48, 49], epitope predictions were undertaken with the signal peptide sequences from the signal peptide database (www.signalpeptide.de), for epitope estimation for several allotypes (Fig 3F). Significantly more peptides with IC_50_ < 500 nM (weak binders) or < 50 nM (strong binders) were identified for B*35:01, B*57 and B*15:01 compared to several members of the B44 supertype. We also examined the prevalence of anchor residues (Fig 3G) and predicted weak and strong binders (Fig 3H) for TAP-dependent and RIT allotypes within human transmembrane sequence datasets (TMbase25, ftp://ftp.ncbi.nih.gov/repository/TMbase/). Similar trends were noted as with signal sequences. Thus, our data support the model that peptide loading in the ER contributes to ER exit of RIT allotypes, which is favored by the increased prevalence of peptides with an appropriate P_2_ residue within signal peptides or transmembrane domains. There is prior evidence for TAP-independent presentation of peptides derived from both of these sources [44–47].

### RIT HLA-B are partially peptide-receptive

**Fig 4:**
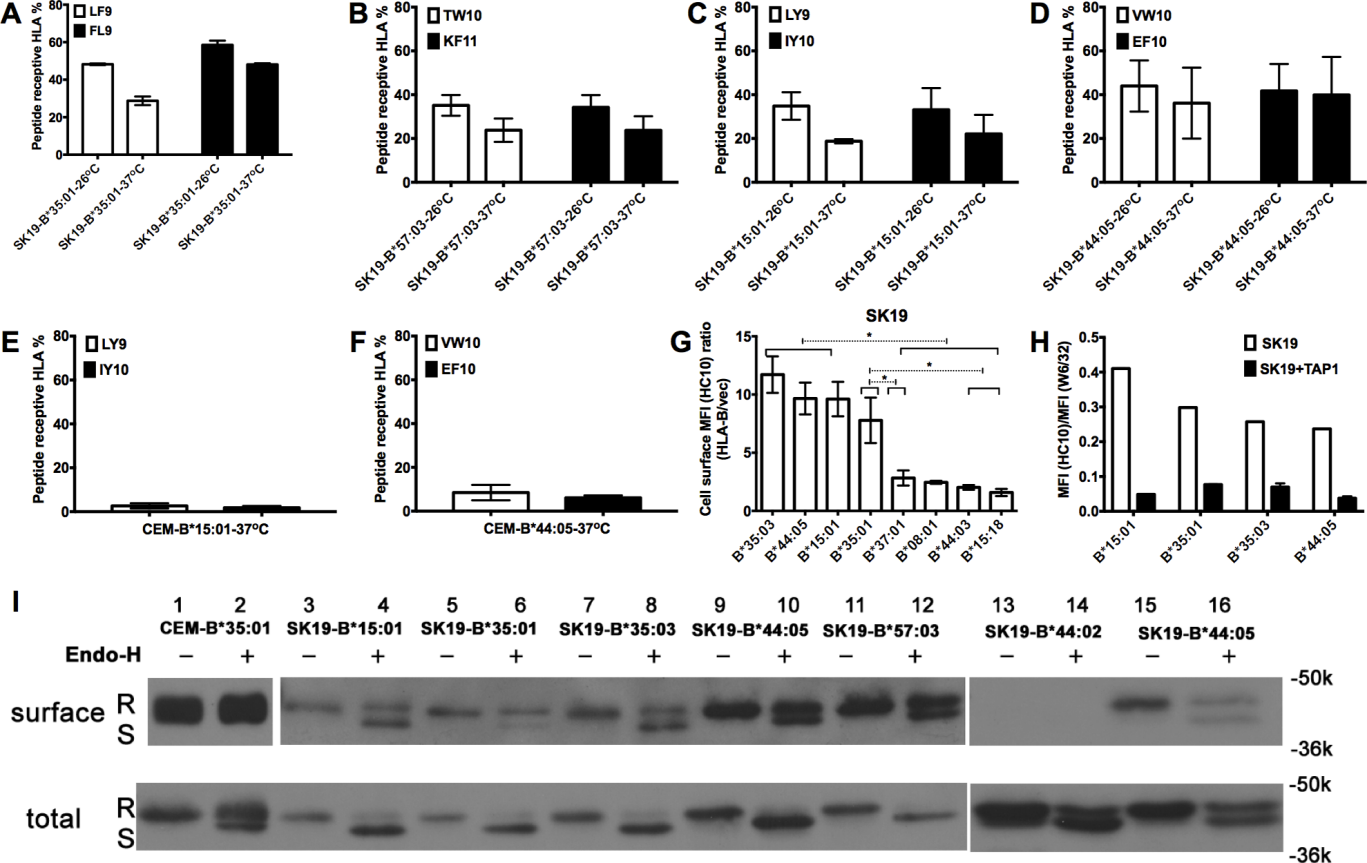
Peptide receptive RIT HLA-B molecules are prevalent on the cell surface of TAP-deficient cells and traffic to the cell surface via conventional and unconventional pathways. (A-D) Peptide binding studies suggest that a significant amount of cell surface RIT HLA-B (B*35:01, B*57:03, B*15:01 and B*44:05) in TAP-deficient cell line SK19 is receptive to exogenous peptides (n = 3 replicates). Peptide receptivity of cell surface HLA-B was assessed as described in methods. Briefly, cells cultured at 26°C or 37°C were incubated with indicated peptides at 26°C for 2h and then incubated in the presence of BFA at 37°C for an additional 2h. The HLA-B signals were quantified by flow cytometry and signals from cells infected with retrovirus lacking HLA-B were subtracted. Peptide receptive HLA-I was quantified as (MFI HLA-I_(+peptide)_–MFI HLA-I_(-peptide)_) / MFI HLA-I_(+peptide)_*100. (E-F) Peptide receptivity of B*15:01 or B*44:05 was assessed following expression in a TAP-sufficient cell line, CEM, as described in methods. Cell surface B*15:01 and B*44:05 are mostly unreceptive to exogenous peptides in these cells. (G) Some cell surface HLA-B is empty as assessed by flow cytometry with HC10, an antibody specific for open HLA class I conformations. HC10-based flow cytometric analysis of selected HLA-B-expressing SK19 cells (obtained as described in Fig 1A, but using the HC10 antibody (n = 5 measurements from a single infection)). Significant differences are indicated (with an asterisk) on the graph (*P*<0.05). Statistical significance is based on an ordinary one-way ANOVA analysis with Fisher’s LSD test. (H) Comparative staining of indicated TAP1-deficient or TAP1-reconstituted SK19 cells (obtained as described in Fig 2A) with W6/32 and HC10 (n = 2 measurements from a single infection). Compared with TAP1-reconstituted SK19 cells, TAP1-deficient SK19 cells expressing RIT HLA-B allotypes showed high HC10 / W6/32 ratios. (I) Cell surface (upper panel) or total HLA-I molecules (lower panel) from SK19-HLA-B or CEM-B*35:01 cells were digested with Endo-H or left undigested, and analyzed by SDS-PAGE and immunoblot described in the method section. R indicates Endo-H resistant HLA-I heavy chain band, and S indicates Endo-H sensitive HLA-I heavy chain band. One representative set of blots from two experiments is shown.

Findings from Fig 3 suggest that signal peptides and protein transmembrane domain-derived peptides could contribute to cell surface HLA-B molecules of RIT allotypes. However, limitation in this pool could result in loading with suboptimal sequences or in partial escape of empty molecules to the cell surface. To test the extent of peptide-receptive cell surface HLA-B, brefeldin A (BFA) decay assays were further conducted in SK19-HLA-B cells that were pre-incubated in the presence or absence of relevant HLA-B-specific peptides. Since anterograde transport is blocked by BFA, and cell surface HLA-I internalization is expected to be more rapid for empty or suboptimally loaded HLA-I [50], the peptide-inducible fraction of the cell surface RIT HLA-B provides an estimate of the fraction of empty or suboptimally loaded cell-surface HLA-B. Based on these analyses, about 30–40% of cell surface RIT HLA-B including B*35:01 (Fig 4A), B*57:03 (Fig 4B), B*15:01 (Fig 4C) and B*44:05 (Fig 4D) are estimated to be expressed in an empty or suboptimally loaded form in TAP1-deficient SK19 cells after overnight culture at 26 ^o^C. Under this condition, empty MHC-I was previously shown to be induced at the cell surface and stabilized by exogenous peptides [25, 50]. Interestingly, even after overnight culture at 37 ^o^C, a condition under which empty MHC-I are generally labile, significant fractions (~20–30%) of the RIT HLA-B allotypes were peptide-inducible (Fig 4A–4D). In contrast, on the surface of TAP-sufficient cells, only a small percentage (~5%) of HLA-B molecules are peptide receptive (Fig 4E and 4F). Thus, TAP-deficiency induces expression of HLA-B that is partially peptide-receptive.

To confirm the presence of suboptimally loaded HLA-B on the cell surface of TAP-deficient cells at 37°C, SK19 cells expressing different RIT HLA-B allotypes were stained with HC10 [51], which detects empty or open HLA-I conformations [52]. Higher levels of HC10-reactive RIT HLA-B allotypes were detectable on the cell surface compared to other HLA-B allotypes (Fig 4G). TAP1 supplementation generally reduced HC10-reactive RIT HLA-B, while simultaneously enhancing the W6/32-reactive forms, contributing to a net decrease in the HC10 / W6/32 ratios (Fig 4H).

### RIT allotypes traffic via conventional and unconventional pathways in TAP-deficient cells

In the classical secretion pathway, HLA-I molecules are transported through the Golgi-network to the cell surface. In this pathway, the quality control machinery will prevent suboptimally loaded HLA-I from migration into the medial Golgi apparatus where proteins are modified and become Endoglycosidase H (Endo-H) resistant. Since a subset of RIT HLA-I molecules are suboptimally loaded under TAP-deficiency conditions (Fig 4A–4H), alternative non-classical secretion pathway might exist to transport suboptimally loaded HLA-I molecules to the cell surface [53]. To address this model, the Endo-H sensitivities of HLA-I molecules in TAP-sufficient CEM and TAP-deficient SK19 cells were assessed. As shown in Fig 4I, most of the HLA-I molecules from either cell surface or total lysate of CEM-B*35:01 cells are Endo-H resistant, indicating that, in the steady state, most HLA-I molecules in CEM cells are mature and they traffic to the cell surface largely through the conventional pathway (Fig 4I). In contrast, a greater fraction of HLA-I molecules from SK19 cells expressing exogenous HLA-B molecules are Endo-H sensitive, suggesting that a larger fraction is ER-retained in SK19 cells compared to CEM cells. Interestingly, following surface biotinylation, a detectable portion of RIT HLA-B molecules on the surface of SK19 cells were found to be Endo-H sensitive, in contrast to the predominantly Endo-H resistant HLA-I of CEM-B*35:01 cells. On the other hand, consistent with flow cytometry data (Fig 1A), cell surface expression of a highly TAP-dependent HLA-allotype B*44:02 was barely detectable following surface biotinylation and immunoblotting (Fig 4I, lanes 13 and 14). These findings suggest a non-Golgi route exists for the trafficking of a subset of HLA-I from the ER to the cell surface of SK19 cells. Taken together, the results reported above suggest that under TAP-deficiency conditions, although a fraction of HLA-B molecules are transported to the cell surface through the conventional pathway, a fraction of RIT-HLA-B molecules follow an alternative non-conventional secretory pathway to reach the cell surface.

### RIT allotypes are resistant to TAP inhibition and inhibitory for NK cell activation

**Fig 5:**
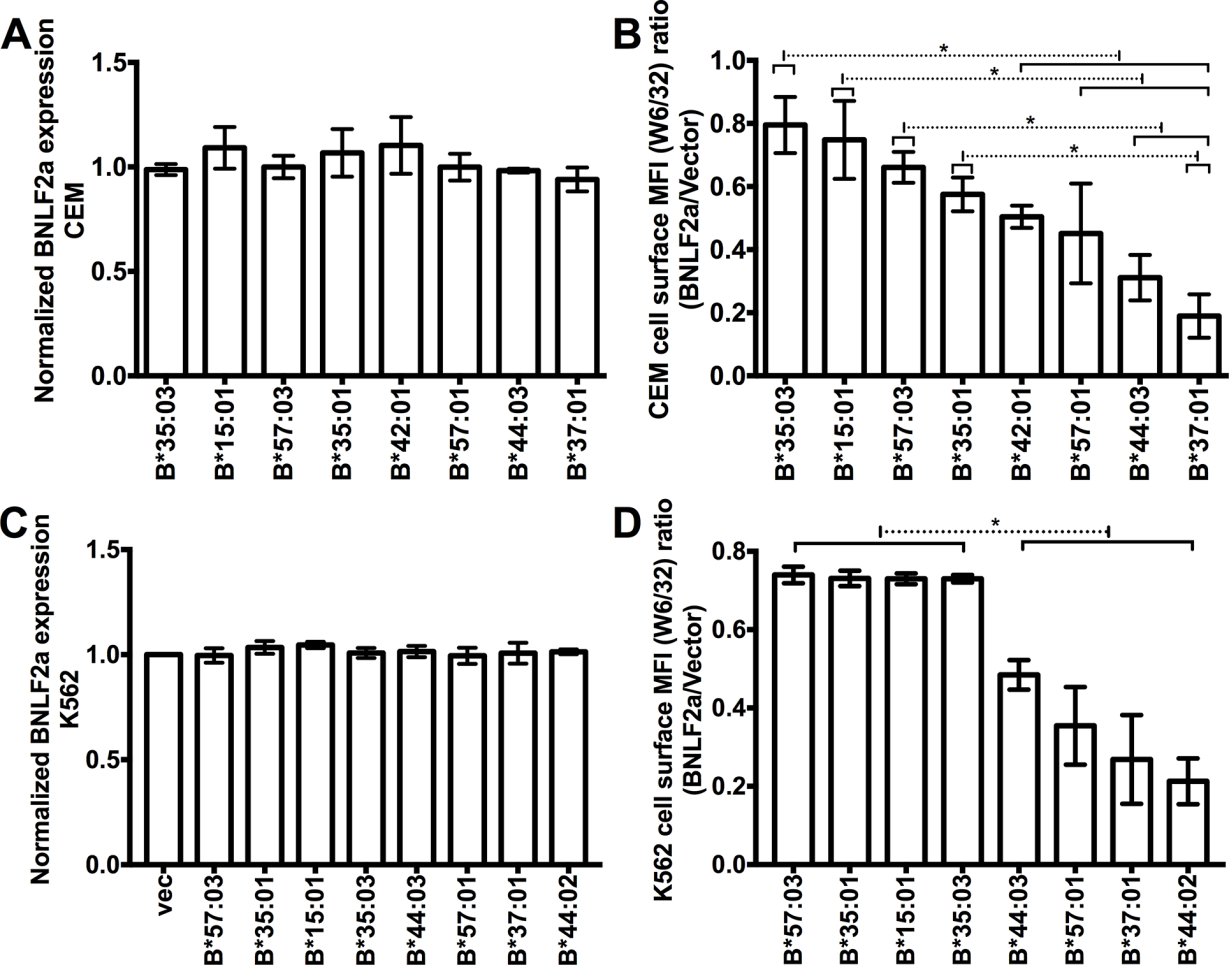
RIT HLA-B allotypes are more resistant to TAP inhibition than non-RIT HLA-B allotypes. (A and B) CEM cells or (C and D) K562 cells expressing different exogenous HLA-B allotypes were infected with a HA-tagged BNLF2a-encoding retrovirus or retrovirus lacking BNLF2a (vector). BNLF2a expression levels were assessed by flow cytometry after intracellular staining with monoclonal anti-HA antibody and normalized to MFI values obtained from CEM or K562 cells lacking exogenous HLA-B (labeled vec) infected with the BNLF2a-encoding retrovirus (A and C). Cell surface HLA-B was measured by flow cytometry after staining with W6/32. The MFI ratios in cells expressing or lacking BNLF2a were calculated (BNLF2a/vector) (n = 3 measurements for each HLA-B expressing CEM cells or K562 cells from one infection with BNLF2a-encoding retrovirus) (B and D).

As an important component of the PLC, TAP becomes a target of immune evasion in many virus-infected cells and tumor cells. For example, the Epstein-Barr virus (EBV)-encoded lytic phase protein BNLF2a acts as a TAP inhibitor by arresting TAP in a transport-incompetent conformation [54]. We examined the effects of BNLF2a on cell surface down-modulation of HLA-B allotypes. Although BNLF2a was transduced to similar levels into CEM cells expressing different HLA-B allotypes (Fig 5A), variable BNLF2a-induced HLA-B down-modulation was observed (Fig 5B), consistent with the prior expression results in TAP-deficient cells (Fig 1A and 1B). Similar results were obtained in K562 cells, which express no endogenous HLA-I (Fig 5C and 5D). Thus, TAP-inhibition has differential effects on cell-surface expression of HLA-B allotypes.

**Fig 6:**
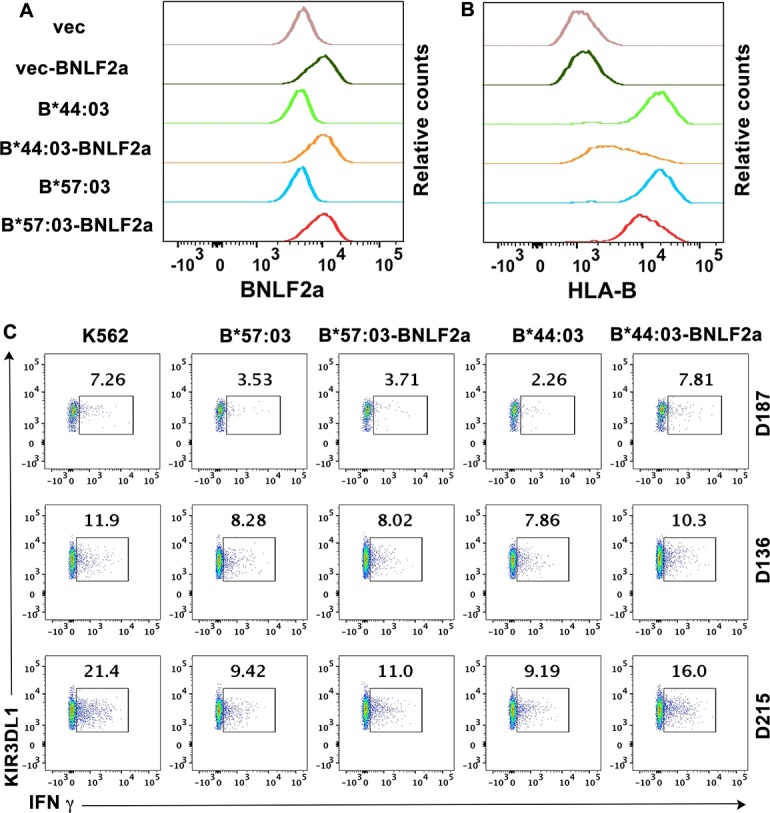
RIT HLA-B allotypes of the Bw4 group are more efficient in inhibiting KIR3DL1^+^ NK cell activation in the presence of the viral TAP inhibitor BNLF2a. HLA-I deficient K562 cells infected with retrovirus encoding exogenous HLA-B*44:03 and HLA-B*57:03 or retrovirus lacking HLA-B (vector) were chosen and further infected with a BNLF2a-encoding retrovirus or retrovirus lacking BNLF2a (vector). Intracellular BNLF2a expression levels (A) and cell surface expression of HLA-B were assessed by flow cytometry (B). HLA-B*44:03 expression is more strongly reduced by BNLF2a than HLA-B*57:03 (one representative experiment of three measurements is shown). (C) K562 cell based NK cell activation assay was performed with PBMCs from three different donors (D187, D136 and D215). CD3^-^CD56^+^KIR3DL1^+^ cells were gated and NK cell activation was assessed by quantifying IFN-γ expressing population. One representative dataset from two experiments is shown.

Cell surface HLA-I with Bw4 epitopes function as inhibitory ligands for NK receptor KIR3DL1 [1]. Down-modulation of HLA-I with Bw4 epitopes can induce NK cell activation via the disengagement of KIR3DL1. We expected that under infection conditions which inhibit TAP function, cells expressing RIT HLA-B would be more resistant to NK cell lysis. For comparisons, we chose K562 cells expressing a highly TAP-dependent allele B*44:03, and a RIT allele B*57:03 and cells subsequently infected with a retrovirus encoding BNLF2a. Cell surface expression of B*44:03 was more strongly decreased by BNLF2a than B*57:03 (Fig 6A and 6B). After co-incubation with K562 cells, NK cells from PBMCs of three donors, D136, D187 and D215, were activated, and expression of IFN-γ was measured (Fig 6C, Column 1). Expression of B*57:03 and B*44:03 in K562 cells strongly inhibits KIR3DL1^+^ NK cell activation (Fig 6C, Columns 2 and 4). In B*44:03 expressing cells (Fig 6C, Column 5) but not B*57:03 expressing cells (Fig 6C, Column 3), KIR3DL1^+^ NK cells activation was increased by BNLF2a expression, consistent with the reduced expression of B*44:03 compared to B*57:03 on the cell surface.

## Discussion

Although the specific epitopes presented by HLA-I allotypes are well studied, the influences of folding and assembly variations among HLA-I allotypes on immunity are poorly characterized. Under normal conditions that are suitable for peptide loading, the effect of folding and assembly variations might not be significant. However, their effects could be amplified under pathological conditions whereby the function of PLC is disrupted by viral infection or tumorigenesis. In support of our prediction, we found that HLA-B allotypes are expressed at different levels on the surface of TAP-deficient or TAP-inhibited cells.

**Fig 7:**
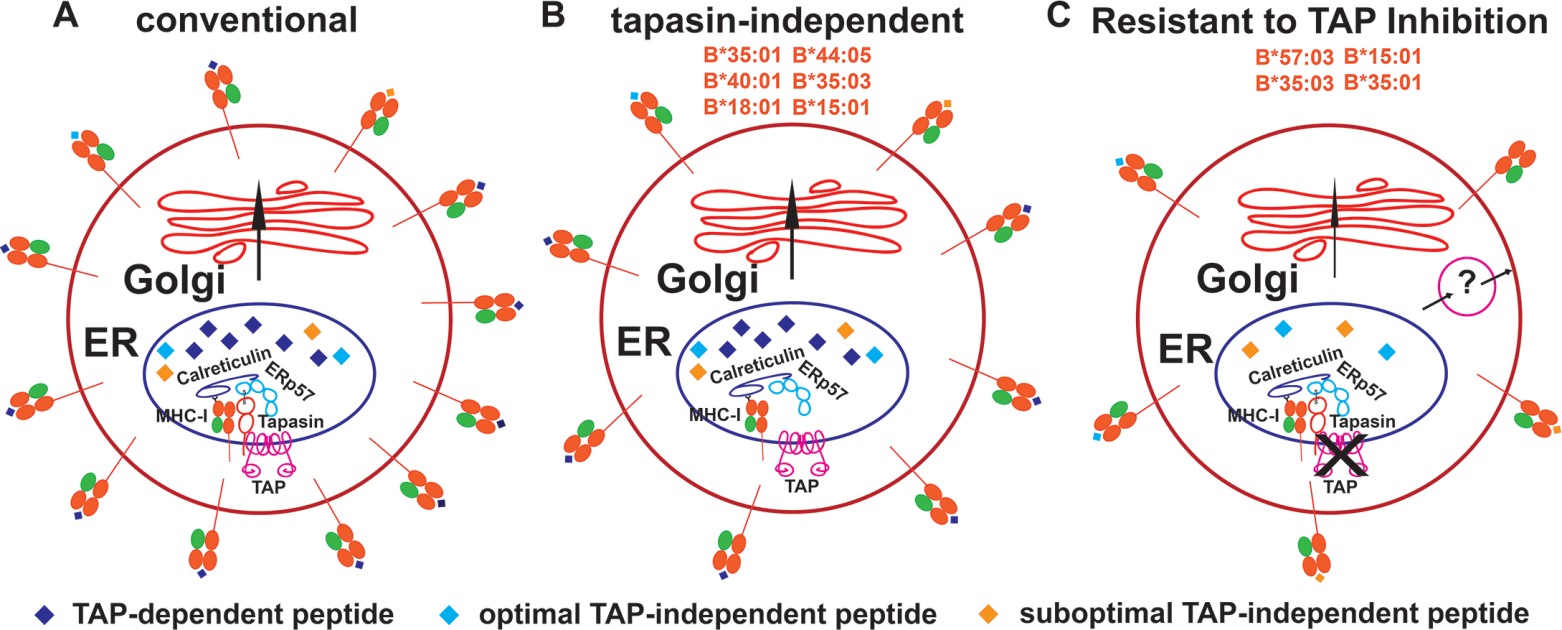
Allele-dependent variations in permissive HLA-B antigen presentation pathways. In the conventional pathway operative in normal cells (A), TAP-dependent peptides are presented by the majority of HLA-B allotypes, although members of the B7 supertype may also present optimal and suboptimal peptides from TAP-independent sources, due to mismatch between their peptide-binding preferences and TAP transport specificity. While expression levels of many allotypes are strongly reduced by tapasin or TAP deficiency, some allotypes are relatively resistant to the deficiency of these factors (B and C). In particular, B*18:01, B*44:05, B*40:01, B*35:01, B*35:03 and B*15:01 are detectable at the highest levels in tapasin-deficient cells (B), based on higher stabilities of the peptide-deficient forms and/or high peptide-loading efficiencies (Ref. 22). B*57:03, B*35:03, B*15:01 and B*35:01 (RIT-HLA-B) are detected at the highest levels under TAP-deficiency conditions (C). The high expression of RIT-HLA-B under TAP-deficiency conditions is mediated by the synergy between their high intrinsic stability, high peptide loading efficiency and generally broader peptide repertoires, particularly for peptides present within unconventional sources such as signal sequences. The strong reduction in the ER peptide levels under TAP-deficiency conditions contributes to the suboptimal loading and cell surface peptide receptivity of RIT-HLA-B. The ER quality control system for the retrieval of suboptimally loaded RIT-HLA-B molecules is imperfect, and alternative (non-Golgi) pathways may exist for transport of suboptimally loaded RIT-HLA-B molecules to the cell surface. RIT-HLA-B allotypes are expected to induce stronger CD8^+^ T cell responses in the context of viruses that inhibit TAP, but weaker NK responses. Conversely, other non-RIT HLA-B are expected to induce stronger NK responses but weaker CD8^+^ T cell responses, following infections with viruses that inhibit TAP.

Our previous findings indicated that, in the absence of peptide, the refolding efficiencies and thermostabilties of HLA-B allotypes are quite variable [12, 22]. Under a tapasin-deficient condition, the capacity for assembly was generally higher for allotypes that had high refolding efficiencies in the absence of a peptide ligand [22]. HLA-I molecules with higher intrinsic stabilities of their peptide-deficient forms were expected to breach ER quality control mechanisms and more readily survive unfavorable assembly conditions such as low peptide supply (TAP-deficiency condition). However, we found that high stability of the peptide-deficient form alone is insufficient to induce the highest level of expression, as exemplified by the intermediate expression level of B*18:01, for which the ER peptide supply is predicted to be highly limiting under TAP-deficiency conditions (Fig 3F and 3H). Based on the findings in this study, we propose the following model: in normal cells when peptide is not limited for most allotypes, cell surface HLA-I molecules are generally loaded with optimal peptides as a result of the abundant peptide pool (Fig 7A). Under a suboptimal condition where the assembly factor tapasin is deficient, the observed expression hierarchy is determined by intrinsic stabilities and peptide loading efficiencies (Fig 7B) [22]. Under a third condition where peptide is highly limited due to TAP inhibition or deficiency (Fig 7C), surface expression of the majority of HLA-B allotypes is strongly reduced. On the other hand, surface expression of RIT allotypes is less affected, because they have high intrinsic stabilities, high peptide loading efficiencies or broader specificities for peptides prevalent within signal sequences or other unconventional sources.

Despite the expected role for peptides from unconventional sources as a determinant of TAP-independent HLA-B expression, many cell surface RIT HLA molecules are suboptimally loaded (Figs 4 and 7C). Suboptimally loaded HLA molecules arise as a result of a limiting supply of peptides in the ER, an imperfect ER quality control system for the retrieval of suboptimally loaded molecules, and alternative (non-Golgi) pathways for transport to the cell surface (Figs 4I and 7C) [53]. The Endo-H sensitive pool of RIT HLA-B is particularly noteworthy (Fig 4), and suggestive of models of peptide loading within a non-conventional secretory pathway for nascent HLA-I molecules, previously described within professional antigen presenting cells (APC) [53]. Other cell types such as melanoma cell lines also appear to have such pathways (Fig 7C). Although components of the PLC are very important for peptide loading to MHC-I molecules, unconventional antigen processing and peptide loading pathways do appear to widely exist (Fig 7B and 7C). Among the tested HLA-B allotypes, B*35:01, B*35:03 and B*15:01 are noteworthy for their high expression when either TAP or tapasin are deficient. Since inhibition of TAP and tapasin is a common evasion strategy used by pathogens and tumors [28, 55] we propose that the folding and assembly characteristics of these allotypes have evolved to allow CD8^+^ T cell-mediated immune surveillance to persist in the face of pathogenic challenges to the conventional pathway.

The B7 supertype is particularly noteworthy for the higher propensity for TAP-independent expression (Fig 3). Allotypes belonging to this supertype bind peptides with proline at P_2_, which are highly disfavored for TAP-mediated transport [35]. In a recent study, we showed that, compared with other HLA-B, those belonging to the B7 supertype tend to be expressed at lower levels in normal human lymphocytes but not monocytes. Taken together with findings in this study, it appears that mismatch between TAP-transporter specificity and HLA-I peptide binding specificity causes suboptimal assembly and expression of allotypes belonging to the B7 supertype in some cell types, but confers an expression advantage under TAP-deficient or TAP-inhibited cells and possibly in professional antigen presenting cells that have specialized antigen acquisition pathways for HLA class I.

While previously it was found that empty MHC-I molecules move to the surface of TAP-deficient cells only at sub-physiological temperature [50], here we show that partially peptide-receptive forms of RIT HLA-B allotypes are expressed on the surface of TAP-deficient cells even at physiological temperature (Fig 4). Duration of HLA-I molecules on the cell surface is dependent on their stabilities [56]. HLA-I molecules with higher stability of their empty forms are also expected to be more stable on the cell surface in their empty forms. On the other hand, for many allotypes, the empty forms will be rapidly internalized and degraded at physiological temperature due to the relative instability. Empty or open MHC-I conformers have been drawing increasing attention in recent years. They are proposed to be ligands for many receptors, including KIR3DS1 [4, 5], KIR3DL2 [57], KIR2DS4 [57] and LILRB2 [58]. Many of the described interactions with open MHC-I involve *in vitro* studies with acid-treated classical HLA-I. The natural prevalence of empty forms of classical HLA-I in cells is thus far poorly characterized. Under normal and TAP-deficiency conditions, RIT allotypes provide a natural source of partially empty class I, and might thus also be more efficient in triggering signals through receptors specific for open HLA-I. Our recent studies indicate that empty HLA-B*35:01 molecules on the cell surface can augment CD8^+^ T cell activation through enhanced engagement with CD8 [12]. Based on those findings, we expect that, under TAP-inhibited conditions, empty forms of all RIT HLA-B can synergize with reduced levels of antigenic peptide-bound versions to facilitate and maintain some level of CD8^+^ T cell surveillance of infections. Thus, although RIT HLA-I molecules may not show specific advantages under optimal antigen presentation conditions, they are expected to be more efficient in presenting TAP-independent peptides to CD8^+^ T cells in infection or tumor conditions involving TAP blockade. Nonetheless, it is important to note that viruses and cancers have developed many other strategies to evade immune recognition, such as the direct down-regulation of HLA-I expression and interference with IFN-γ signaling (for example, [59]). Thus, cells expressing RIT HLA-B could still escape immune surveillance under other different pathogenic conditions.

T cell epitopes associated with impaired antigen presentation (TEIPP) [60, 61] are known to emerge under conditions of inhibited antigen presentation, including TAP-deficiency conditions. In fact, it is reported that CD8^+^ T cells responsive specifically to TAP-inhibited cells are widely prevalent in the human blood probably due to the prevalence of viruses that encode TAP inhibitors such as EBV, CMV and HSV [62]. Given the high expression levels and suboptimal peptides, RIT HLA-B molecules may contribute dominantly to the HLA-B-restricted CD8^+^ T cell repertoire against TEIPP (including both self-peptides and viral epitopes) under conditions where TAP expression is inhibited or TAP function is suppressed, an area for further assessment. Moreover, the prevalence of RIT HLA-B molecules might be a reason that there is only mild immunodeficiency in TAP deficient humans [63], and RIT-HLA-I may be the dominant antigen presenting alleles in these patients.

In conclusion, it is well recognized that pathogens have developed strategies to escape cytotoxic T cell surveillance by, for example, disrupting HLA-I assembly pathways [28, 29]. It is now apparent that HLA-I molecules have also evolved to assemble via distinct pathways, which are allotype dependent, as a way to counter pathogen evasion strategies that target the conventional assembly pathway (Fig 7). Thus, the textbook-defined HLA-I assembly pathways are not fully applicable to all allotypes. In this study, we demonstrate that 15% of tested HLA-B allotypes are resistant to inhibition or deficiency in TAP, which is considered a central source of peptides for HLA-I assembly. Cell surface expression of several HLA-B allotypes is readily observable under TAP-deficiency conditions, and relates to HLA-B intrinsic stabilities, peptide loading efficiencies, peptide binding preferences and unconventional secretory pathways. Thus, TAP-independent pathways of antigen acquisition are quite broadly prevalent. RIT HLA-B molecules are expected to confer immune recognition advantages for the CTL response under TAP-inhibited conditions, via the mechanisms outlined above. Conversely, when TAP function is blocked, HLA-B allotypes with Bw4 epitopes that are strongly down-modulated confer induced abilities to mediate NK activation, via reduced KIR3DL1^+^ NK cell binding (Fig 6). Overall, the findings in this study point to important functional distinctions within the HLA-B locus that relate back to intrinsic structural features of the proteins and their intracellular assembly characteristics.

## Materials and methods

### Ethics statement

Blood was collected from consented healthy donors for functional studies in accordance with a University of Michigan IRB approved protocol (HUM00071750). All donors provided informed written consent.

### Cell lines

Human melanoma cell line SK-mel-19 (SK19) [37] (obtained from the laboratory of Dr. Pan Zheng), fibroblast cell line STF1 [38] (obtained from the laboratory of Dr. Henri de la Salle), cervical cancer cell line Hela (obtained from the laboratory of Dr. Oveta Fuller) and ecotropic virus packaging cell line BOSC (obtained from the laboratory of Dr. Kathleen Collins) were grown in DMEM (Life Technologies) supplemented with 10% (v/v) FBS (Life Technologies) and 1× Anti/Anti (Life Technologies) (D10). T4-lymphoblastoid cell line CEM-ss (CEM) cells (obtained from the laboratory of Dr. Kathleen Collins) and chronic myelogenous leukemia cell line K562 cells (obtained from ATCC; CCL-243) were grown in RPMI 1640 (Life Technologies) supplemented with 10% (v/v) FBS, 1× Anti/Anti, 2 mM glutamine (Life Technologies) and 10 mM HEPES (Life Technologies) (R10).

### Antibodies

The following monoclonal antibodies were used in this study: Pacific Blue-conjugated anti-human CD3 (clone UCHT1; BioLegend), PE-Cy7-conjugated anti-human CD56 (clone CMSSB; eBioscience), FITC-conjugated anti-human KIR3DL1 (clone DX9; BioLegend), Alexa Fluor 700 conjugated anti-human IFN-γ (clone B27; BioLegend), purified anti-HA.11 (Clone 16B12; BioLegend), anti-BiP (Clone C50B12; Cell Signaling Technology), anti-GAPDH (Clone 14C10; Cell Signaling Technology) and anti-vinculin (Clone E1E9V; Cell Signaling Technology). Dead cells were excluded from flow cytometric analyses with 7-amino-actinomycin D (7-AAD; BD Biosciences) or the amine-reactive dye Aqua (405nm, Life Technologies). HLA-I antibodies W6/32, HC10 and 171.4 were produced in the University of Michigan Hybridoma Core. The TAP1 antibody 148.3 was kindly gifted by Dr. Robert Tampé.

### Viruses and cell infections

All HLA-B alleles in the retroviral vector LIC pMSCVneo were prepared as described previously [22]. HA-tagged versions of HLA-B*35:01, B*35:03, B*57:01, B*44:02 and B*4405 were prepared as described previously [64, 65]. To prepare HA-tagged versions of HLA-B*15:01, B*44:03, B*57:03 and B*58:02, corresponding clones from pMSCVneo [22] were digested with NaeI and XhoI to prepare the 3′ regions of these HLA-B (encoding the portion of the protein downstream of the signal sequence). The B*35:01 signal sequence plus HA-tag was isolated by EcoRI and NaeI digestion of HA tagged B*35:01. Finally, the HLA-B*15:01, B*44:03, B*57:03 and B*58:02 NaeI–XhoI fragments and the EcoRI-NaeI fragment from HA-B*35:01 were ligated into pMSCVneo (cut with EcoRI and XhoI) in a three-way ligation. Retroviruses were generated using BOSC cells and used to infect SK19, STF1, Hela, CEM or K562 cells. Cells were infected with retroviruses encoding the HLA-B molecules, selected by treatment with 1 mg/ml G418 (Life Technologies), and maintained in 0.5 mg/ml G418. Exogenous HLA-I expression was verified by immunoblotting analyses of cell lysates using the mouse anti-human monoclonal antibody 171.4 or anti-HA and secondary antibodies GαM-HRP (Jackson ImmunoResearch Laboratories) or GαM-IRDye 800CW (LI-COR Biosciences). SK19 cells expressing exogenous HLA-B molecules were infected with the human TAP1-encoding retrovirus and selected by treatment with 1 μg/ml puromycin (Sigma-Aldrich), and cells were maintained in 0.5 μg/ml puromycin. TAP1 expression in SK19 cells was verified by immunoblotting analysis of cell lysates using mouse anti-human TAP1 monoclonal antibody 148.3 [66] and secondary antibodies GαM-HRP or GαM-IRDye 800CW. The Western blots were developed for chemiluminescence using the GE Healthcare ECL Plus kit or scanned for IRDye fluorescence using Odyssey System (LI-COR Biosciences). CEM and K562 cells expressing exogenous HLA-B molecules were infected with the BNLF2a-encoding retrovirus and selected by treatment with 1 μg/ml puromycin (Sigma-Aldrich), and cells were maintained in 0.5 μg/ml puromycin. MSCV-N BNLF2a was a gift from Dr. Karl Munger [67] (Addgene plasmid # 37941). BNLF2a expression was verified by intracellular staining with primary antibody anti-HA and secondary antibody PE-conjugated goat anti-mouse IgG (GαM-PE, Jackson ImmunoResearch Laboratories).

### TAP1 knock down in Hela cells

TAP1 was knocked-down in Hela cells by using the CRISPR/Cas9 system based TAP1 Double Nickase Plasmid from Santa Cruz Biotechnology according to manufacturer’s protocol. Puromycin selection and limiting dilution was subsequently undertaken to obtain monoclonal TAP1-KD cell lines. TAP1 knockdown was verified by immunoblotting analysis of cell lysates using anti-TAP1 antibody 148.3 [66] and secondary antibodies GαM-HRP (goat anti-mouse horse radish peroxidase) and by intracellular staining with 148.3 [66] and secondary antibody GαM-PE. HLA-B alleles were expressed in Hela or Hela-TAP1-KD cells using the method described above.

### Flow cytometric analysis to assess MHC-I cell surface expression

A total of 1×10^5^−1×10^6^ cells were washed with FACS buffer (phosphate-buffered saline (PBS), pH 7.4, 1% FBS) and then incubated with W6/32 or HC10 antibodies at 1:250 dilutions or anti-HA at 1:50 dilution for 30–60 min on ice. Following incubation, the cells were washed three times with FACS buffer and incubated with GαM-PE or GαM-PE-Cy7 at 1:250 dilutions for 30–60 min on ice. The cells were then washed three times with FACS buffer and analyzed using a BD FACSCanto II cytometer. The FACS data were analyzed with FlowJo software version 10.0.8 (Tree Star, San Carlos, CA). Data are deposited in the Dryad repository: http://dx.doi.org10.5061/dryad.m4862mk [68].

### Peptide receptivity assessments

The night before the experiment, cells were moved to 26°C or kept at 37°C. The next day, cells were washed with PBS, and the medium (containing 100 μM peptide where indicated) was added and cells were incubated at 26°C for 2h. Cells were then incubated at 37°C in the presence of 20 μg/ml brefeldin A (BFA) for an additional 2h and then harvested. The HLA-B signals were quantified by flow cytometry after staining with W6/32 and subtracting signals obtained from cells infected with a retrovirus lacking HLA-B. Peptide receptive HLA-I was quantified as (MFI HLA-I_(+peptide)_–MFI HLA-I_(-peptide)_) / MFI HLA-I_(+peptide)_*100 and averaged across 3–4 independent measurements for each condition. Peptides used (S1 Table) were B*57:03-restricted epitopes TSTLQEQIGW (TW10) and KAFSPEVIPMF (KF11), B*44:05-restricted epitopes VEITPYKPTW (VW10) and EEFGRAFSF (EF10), B*15:01-restricted epitopes LEKARGSTY (LY9) and ILKEPVHGVY (IY10) and B*35:01-restricted epitopes FPVRPQVPL (FL9) and LPSSADVEF (LF9) [64]. All peptides were purchased from peptide 2.0 (Chantilly, VA, USA). All peptides are in the IEDB database except self-peptide LF9.

### Endo-H sensitivity assay

Cell surface proteins were biotinylated by incubating cells with 2mM EZ-Link NHS-PEG4-Biotin (Thermo Scientific) in PBS for 10 min at room temperature followed by three washes in PBS. After washing, labeled cells were lysed in lysis buffer (1× PBS, 1 mM phenylmethylsulfonyl fluoride, and 1% Triton X-100) for 1h on ice. The lysates were centrifuged at 13,000 g to remove cell debris. Biotinylated proteins were bound to streptavidin conjugated beads for 2 h at 4°C. Beads were washed three times with lysis buffer, and boiled for 10 min in the presence of denaturing buffer. As controls, total cell lysates were directly boiled for 10 min in denaturing buffer. The materials obtained from the beads and total cell lysates were split into two equal aliquots and one of the aliquots was digested with Endo-H (New England Biolabs) according to the manufacturer’s protocol. HLA-I molecules were separated by SDS-PAGE and then immunoblotted using the mouse anti-human monoclonal antibody 171.4.

### NK cell activation assay

Fresh blood collected from donors was subjected to centrifugation over a Ficoll-Paque Plus (GE Healthcare Life Sciences) density gradient, washed twice with PBS + 2% FBS and resuspended in R10. Isolated PBMCs were cryopreserved in Recovery Cell Culture Freezing Medium (Life Technologies). IFN-γ expression in NK cells was detected by intracellular cytokine flow cytometry. Briefly, frozen PBMCs (2 × 10^5^ cells/well) were incubated with K562 cells expressing or lacking HLA-B molecules at 1:1 (PBMC:K562) ratio in 200 μL complete media in 96-well U-bottom plates. GolgiPlug (containing brefeldin A, BD Biosciences) was added at 1:1000 1h later. After incubation for an additional five hours, cells were stained with Pacific Blue-conjugated anti-CD3, PE-Cy7-conjugated anti-CD56 and FITC-conjugated anti-KIR3DL1 mAbs for 30 minutes at 4°C, fixed in 4% paraformaldehyde for 10 minutes at room temperature, and permeabilized with 0.2% saponin for 10 minutes. Cells were then stained with Alexa Fluor 700-conjugated anti-IFN-γ for 30 minutes at 4°C and analyzed by flow cytometry.

### Statistical analysis

Statistical analyses (ordinary one-way ANOVA analysis with Fisher’s LSD test) were performed using GraphPad Prism version 7.

## Supporting information

S1 Table Peptides used in this study. (DOCX)

S1 Fig HLA-I expression levels assessed by immunoblots, related to Fig 1. Total HLA-I expression levels in SK19 cells (A) or STF1 cells (B) expressing indicated exogenous HLA-B were tested by fluorescence-based immunoblotting with the heavy chain–specific 171.4 antibody. (C) Total HA-tagged HLA-I expression levels in SK19 cells expressing indicated exogenous HLA-B were tested by chemiluminescence-based immunoblotting with HA antibody. Vinculin was used as an internal control. Representative immunoblots of indicated cell lysates are shown. A total of 50 μg cell lysate was loaded in each lane.
(TIF)

S2 Fig BiP expression levels in SK19 and STF1 cells assessed by immunoblots, related to Fig 1. BiP expression levels in SK19 cells (A) or STF1 cells (B) expressing indicated exogenous HLA-B or the infection control lacking HLA-B (vec) were tested by immunoblotting. Cells treated with thapsigargin (1 μM, O/N), which is a widely used as an UPR inducer, were used as positive controls. GAPDH expression was tested in parallel as internal control. 5, 10 or 20 μg of cell lysate was loaded in each lane.
(TIF)

S3 Fig TAP1 expression levels assessed by immunoblots, related to Fig 2. TAP1 expression levels in SK19 cells or SK19 cells expressing indicated exogenous HLA-B (A) or HA-tagged exogenous HLA-B (B) were tested by immunoblotting with TAP1 specific antibody 148.3. GAPDH was used as internal control. Representative immunoblots of indicated cell lysates are shown. A total of 50 μg cell lysate was loaded in each lane.
(TIF)
