# Supplementary material for: Selected HLA-B allotypes are resistant to inhibition or deficiency of the transporter associated with antigen processing (TAP)
Source: PLoS Pathog. 2018 Jul 11;14(7):e1007171. doi: 10.1371/journal.ppat.1007171 (PMC6056074; doi:10.1371/journal.ppat.1007171)
Supplement: S1 Table — (DOCX) [file ppat.1007171.s001.docx]

**S1 Table. Peptides used in this study.**

| **Peptide sequence** | **Abbreviation** | **Allele** | **Source** |
| --- | --- | --- | --- |
| TSTLQEQIGW | TW10 | B*57:03 | HIV 1 gag p24 |
| KAFSPEVIPMF | KF11 | B*57:03 | HIV 1 gag p24 |
| VEITPYKPTW | VW10 | B*44:05 | Human herpes virus 4 EBNA-3B nuclear protein |
| EEFGRAFSF | EF10 | B*44:05 | Human HLA-DPA1 |
| LEKARGSTY | LY9 | B*15:01 | Human herpes virus 4 EBNA-3A nuclear protein |
| ILKEPVHGVY | IY10 | B*15:01 | HIV 1 RT |
| FPVRPQVPL | FL9 | B*35:01 | HIV 1 Nef |
| LPSSADVEF | LF9 | B*35:01 | Human tyrosinase |
