# Supplementary figures and images for: Selected HLA-B allotypes are resistant to inhibition or deficiency of the transporter associated with antigen processing (TAP)
Source: PLoS Pathog. 2018 Jul 11;14(7):e1007171. doi: 10.1371/journal.ppat.1007171 (PMC6056074; doi:10.1371/journal.ppat.1007171)

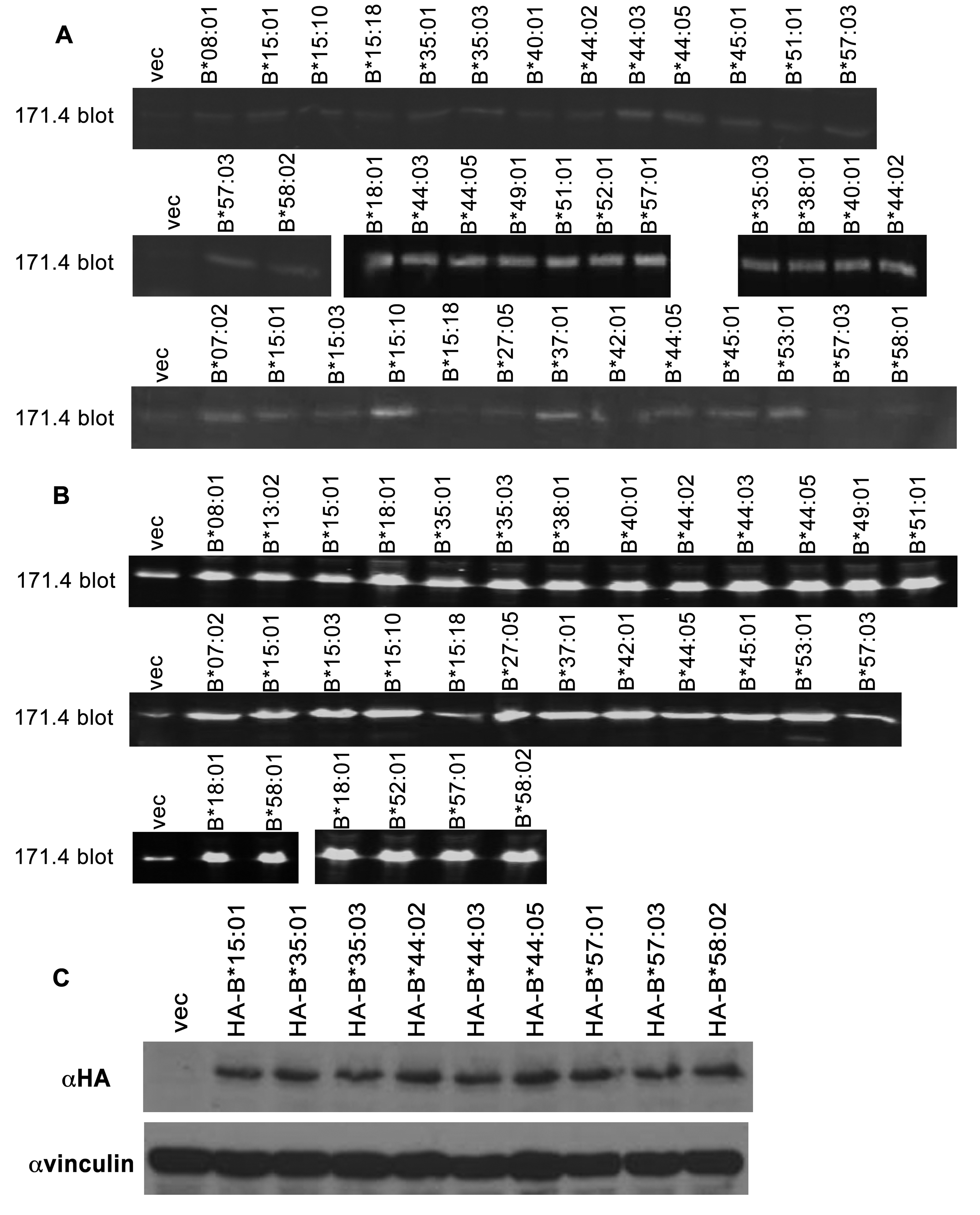

Supplement: S1 Fig — Total HLA-I expression levels in SK19 cells (A) or STF1 cells (B) expressing indicated exogenous HLA-B were tested by fluorescence-based immunoblotting with the heavy chain–specific 171.4 antibody. (C) Total HA-tagged HLA-I expression levels in SK19 cells expressing indicated exogenous HLA-B were tested by chemiluminescence-based immunoblotting with HA antibody. Vinculin was used as an internal control. Representative immunoblots of indicated cell lysates are shown. A total of 50 μg cell lysate was loaded in each lane. (TIF) [file ppat.1007171.s002.tif]

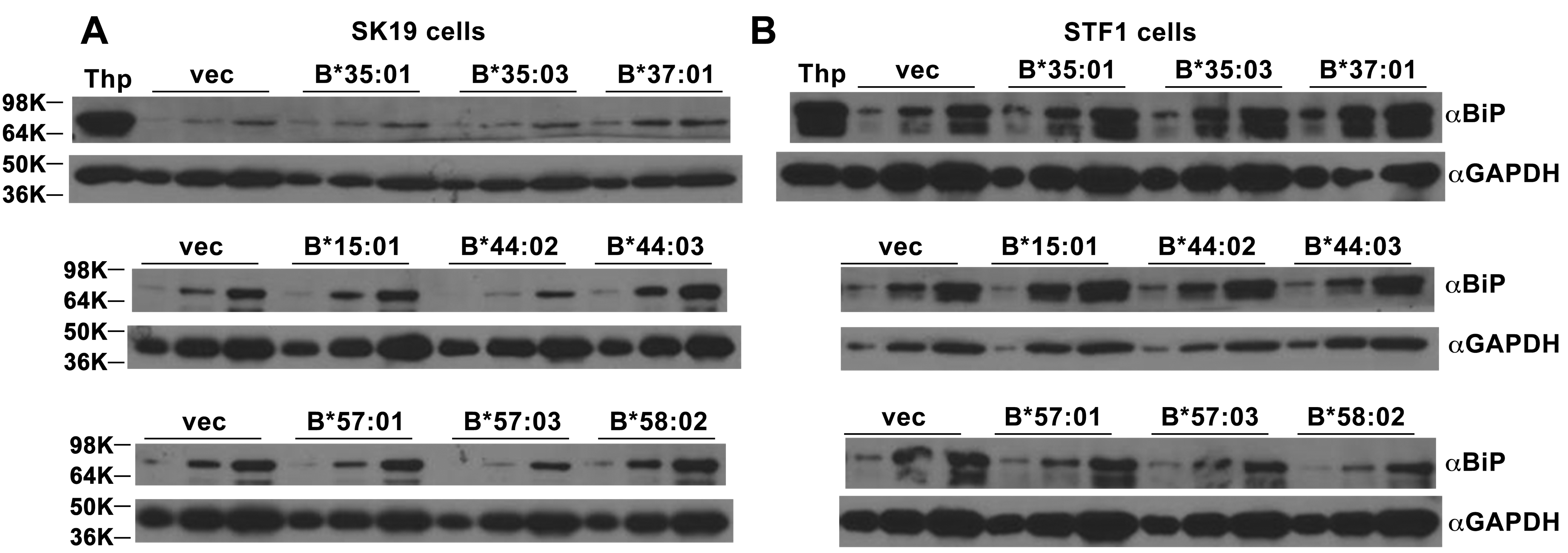

Supplement: S2 Fig — BiP expression levels in SK19 cells (A) or STF1 cells (B) expressing indicated exogenous HLA-B or the infection control lacking HLA-B (vec) were tested by immunoblotting. Cells treated with thapsigargin (1 μM, O/N), which is a widely used as an UPR inducer, were used as positive controls. GAPDH expression was tested in parallel as internal control. 5, 10 or 20 μg of cell lysate was loaded in each lane. (TIF) [file ppat.1007171.s003.tif]

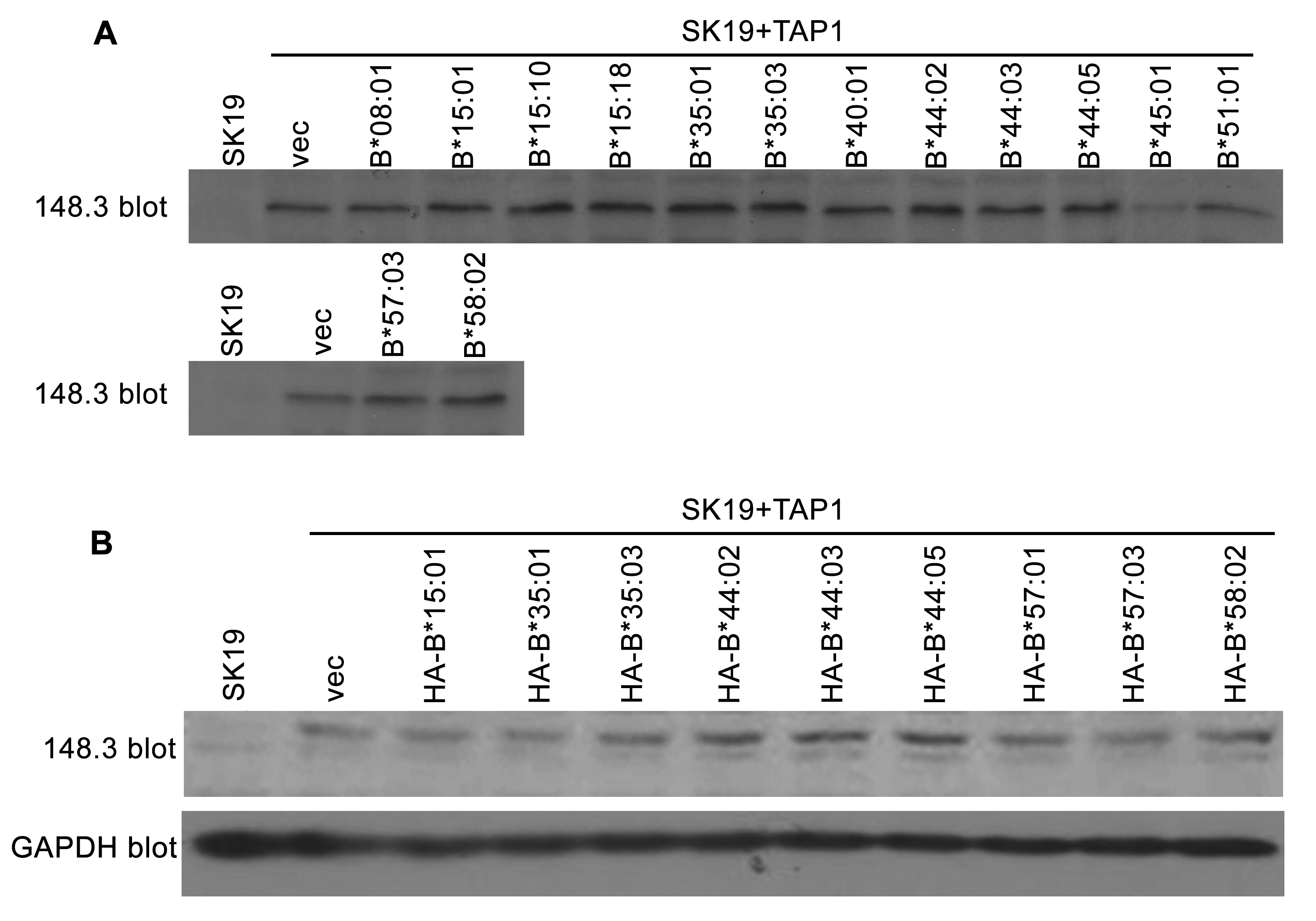

Supplement: S3 Fig — TAP1 expression levels in SK19 cells or SK19 cells expressing indicated exogenous HLA-B (A) or HA-tagged exogenous HLA-B (B) were tested by immunoblotting with TAP1 specific antibody 148.3. GAPDH was used as internal control. Representative immunoblots of indicated cell lysates are shown. A total of 50 μg cell lysate was loaded in each lane. (TIF) [file ppat.1007171.s004.tif]
